# A business approach to transforming public health supply systems

**DOI:** 10.1186/2052-3211-7-S1-O1

**Published:** 2014-12-17

**Authors:** Ellen T Tompsett, Alan Bornbush, Todd Dickens, Carolyn Hart, Chris Wright

**Affiliations:** 1 Reproductive Health Supplies Coalition, Washington DC, USA; 2 Path, Washington DC, USA; 3 JSI, Washington DC, USA

## Background

While much attention is on relatively near-term goals (eg Family Planning (FP) 2020), there is growing interest in and a need to address longer term, in-a-generation "end games" (e.g., to 2035), as well as post-2015 Millennium Development Goals (MDGs). The health supply systems of today need to prepare themselves to take advantage of future trends and opportunities. The next generation will see a number of changes (known and unknown) that will both challenge the ability of public health supply systems to function effectively as they are currently designed and create opportunities for increased efficiency.

## Method

Written with key decision-makers and leaders in mind, this paper draws from the accumulated expertise of the authors along with desk research based on completed case studies. The paper was then peer reviewed by select supply chain experts.

## Results

The people responsible for public health supply systems must change or expand the perspectives of their own roles as well as the mission and composition of the supply systems they oversee or support. In making this shift there are three important guiding principles to keep in mind:

1. A government’s role is one of stewardship in achieving common development goals.

2. Recognize the multiplicity of players and diversity of supply chain options that can now contribute to meeting improved public health outcomes.

3. Understand the broader public health outcomes that supply chains should be designed to support.

## Discussion

Every country context is different, and as such there is no single system design standard, but there are several key points that can be embraced by governments as they look to better position their health supply chains for the future: know your business, focus on what only you can do, learn from the commercial sector, pursue diversity.

## Lessons learned

Change will happen; it is inevitable. The successful stewards of health systems will take charge and lead the change, leverage the multiplicity of supply chain actors, and define a new vision for getting products to people to support the overall goal of improving health outcomes.

